# Antioxidants Profiling of By-Products from Eucalyptus Greenboards Manufacture

**DOI:** 10.3390/antiox8080263

**Published:** 2019-08-01

**Authors:** Maria Celeiro, J. Pablo Lamas, Rosa Arcas, Marta Lores

**Affiliations:** 1 Laboratory of Research and Development of Analytical Solutions (LIDSA), Department of Analytical Chemistry, Nutrition and Food Science, Faculty of Chemistry, Campus Vida, Universidade de Santiago de Compostela, E-15782 Santiago de Compostela, Spain; 2 Betanzos HB, Betanzos-Santiago Road, Km 3, E-15300 Betanzos, A Coruña, Spain

**Keywords:** bioactive compounds, wood industry by-products, antioxidants, analytical characterization

## Abstract

Three different by-products from the *Eucalyptus* wood industry have been exhaustively characterized to find compounds with antioxidant properties. The industrial process to manufacture *Eucalyptus* greenboards is distinguished by using just wood and water, which converts the generated by-products in a highly attractive source of bioactive compounds that are originally in the raw material. The studied by-products were: the screw water, derived from the washing of the wood chips; the condensates, obtained after the evaporation and further condensation of the screw water; and finally, the concentrate of eucalyptus. For all of them and for their derived organic extracts, the total polyphenols content (TPC) and antioxidant activity (AA) have been evaluated. The chromatographic fingerprints, based on gas chromatography-mass spectrometry (GC-MS), and liquid chromatography-tandem mass spectrometry (LC-MS/MS) have been obtained to identify the main extractable organic wood components. Besides, solid-phase microextraction (SPME) has been employed to characterize the most volatile compounds. Significant differences were observed for the chromatographic profiles of the studied by-products. Up to 48 and 30 different compounds were identified in the screw water, and condensate by-products, respectively; whereas the highest number of compounds, up to 72, have been identified in the organic extracts derived from the concentrate of Eucalyptus, highlighting the presence of monoterpenes, sesquiterpenes, polyphenols, and other bioactive compounds with antioxidant properties. Therefore, these by-products could be exploited to obtain natural extracts with added value which could be reused in the food, cosmetic or pharmaceutical industry, reducing the environmental impact of the industrial activity.

## 1. Introduction

All vascular plants produce polyphenols, terpenes and/or alkaloids as secondary metabolites involved in their defence systems. Many of these compounds could be regarded as high-added-value compounds due to their recognized bioactivities, including antimicrobial, antioxidant and even antitumor actions. The trees belonging to the genus *Eucalyptus* contain a wide variety of such bioactive compounds in leaves [1,2,3], stumps [4], bark [5] or fractions obtained from their wood [6]. Indeed, *Eucalyptus globulus* has traditionally been used as a medicinal plant, and Eucalyptus oils, prepared by steam distillation, are also used as flavouring and fragrances [2]. Even recently, three new polyphenolic acids with antivirus activity has been isolated from the leaves of *Eucalyptus citriodora* [7].

Eucalyptus wood is also one of the main raw materials in the timber sector, particularly in specific geographical areas such as the Iberian Peninsula, where the 1.4 million ha occupied by the eucalyptus tree accounts for 7% of the world’s eucalyptus plantations, 53% taking into account only the white eucalyptus (*Eucalyptus globulus*) [8]. Most of this wood is used in the manufacture of paper pulp and, consequently, the wastes generated in this industrial activity, as the black liquor produced from the sulphate process when digesting wood into pulp, are deeply and profusely studied [9,10,11,12,13]. There are also several approaches for the valorization of these wastes [13,14,15] and even for the recovery of some bioactive compounds from the large amounts of bark derived from the log debarking, the first operation in the pulping industrial plants [5].

An alternative way to process eucalyptus wood is the production of high-density wooden fibreboards, using wood and water as the only raw materials and taking advantage of the thermoplastic properties of the lignin as a natural adhesive instead of using artificial glues. The final products are the so called ‘greenboards’ that do not emit formaldehyde and can be considered an ecological material, because its composition makes it a biodegradable material, allowing its re-exploitation as a natural heat source to produce energy at the end of its service life. Even more interesting is that some of the by-products generated in the production process contain a huge percentage of the initial load of bioactive compounds from the original trees. The straightforward idea that emerges from this situation is the simultaneous possibility of find alternatives to the non-renewable sources of bioactive chemicals and to valorize the industrial by-products of the greenboards manufacturing process.

However, something else is needed: to know the composition of the different by-products of the process, for which chromatographic techniques (both gas chromatography, GC and liquid chromatography, LC) coupled to mass spectrometry (MS) emerge as the most useful tools to address this issue. Additionally, we must bear in mind that, unlike black liquor and other residues from the production of paper pulp, the target by-products from the greenboards production have never been chemically characterized.

Therefore, the aim of this work is first to obtain the chromatographic profiles of three different *Eucalyptus* by-products, named screw waters, condensates and concentrate, in order to chemically characterize them as deeply as possible, on the basis of their organic compounds composition; and, then, select those compounds that may be of most interest for their properties or bioactivities. In parallel, the possibility of extracting these compounds of interest with selected organic solvents has also been studied, initially on a laboratory scale, in order to evaluate the potential application of the phytochemicals enriched extracts in different sectors, mainly cosmetics and food. In addition, basic physic-chemical parameters, indicative indices of their bioactive properties, together with stability studies have also been carried out. Finally, the most interesting properties of some of the bioactive compounds identified are also discussed.

## 2. Materials and Methods

### 2.1. Reagents and Materials

Methanol and water (MS grade) were provided by Scharlab (Barcelona, Spain), ethanol (EtOH) absolute (>99.8%) was provided by VWR (Leicestershire, England), ethyl lactate was supplied by Fluka Analytical (Steinheim, Germany), and formic acid (>99%) by Merck (Darmstadt, Germany). Folin–Ciocalteu phenol reagent, 2,2-diphenyl-1-picrylhydrazyl (DPPH), and gallic acid (99%) were obtained from Sigma-Aldrich (Steinheim, Germany). Sodium carbonate (Na_2_CO_3_) was provided by Panreac (Barcelona, Spain), and sodium sulphate anhydrous (Na_2_SO_4_) was provided by Carlo Erba Reagents (Peypin, France).

Commercial 65 µm polydimethylsiloxane/divinylbenzene (PDMS/DVB) fibre housed in manual SPME holders was obtained from Supelco (Bellefonte, PA, USA). The fibre was conditioned as recommended by the manufacturer (270 °C for 30 min), inserting it in the GC injector with carrier gas flow. Protocatechuic acid (96.5%), 3,4-hydroxybenzaldehyde (97%), chlorogenic acid (99%), and 4-hydroxybenzaldehyde (97%) were supplied by Sigma-Aldrich (Steinheim, Germany).

### 2.2. Studied By-Products

All the studied by-products (Eucalyptus screw water, condensates, and the concentrate) were provided by the wood board industry Betanzos HB (Betanzos, Galicia, NW Spain), specialized in the elaboration of high-density fiberboards employing a green industrial procedure based on high-pressure pressing employing water without chemicals additives. All the raw material samples were collected in 2.5 L plastic bottles, and they were kept (less than 48 h) at room temperature and protected from light until their analysis.

**Figure 1:**
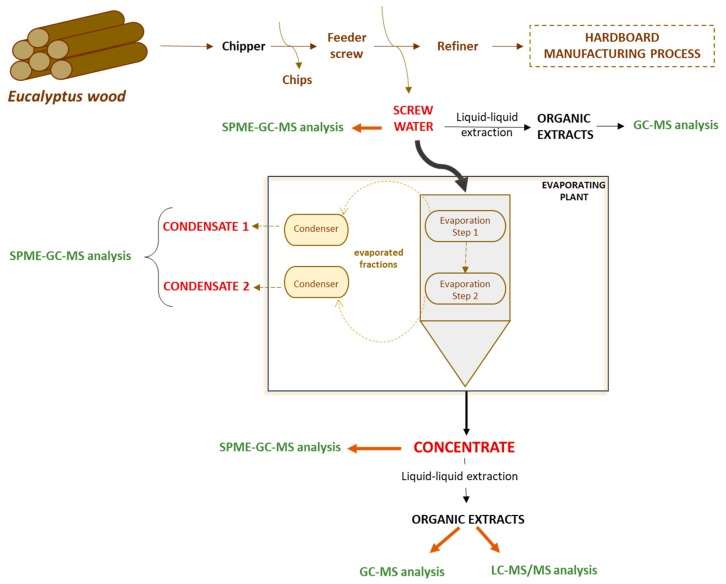
Outline of the Eucalyptus greenboard production process showing the generated by-products, their derived organic extracts and the chromatographic strategies used for their characterization.

Figure 1 summarizes the industrial procedure, indicating the origin of the three studied by-products, and the employed chromatographic technique for their characterization.

The screw water comes from washing the Eucalyptus wood. The condensates by-products were obtained from the evaporation and further condensation of the screw water. Two different condensates were obtained: Condensate 1 from a first evaporation/condensation step, and Condensate 2, obtained as result of a second evaporation and condensation. Finally, the residual phase that remains after the second evaporation step is the known as Eucalyptus concentrate.

### 2.3. Liquid-Liquid Extraction

Ethanol and ethyl lactate have been employed as solvents to obtain the wood by-products (concentrate of Eucalyptus and Eucalyptus screw water) extracts. Ethanol was selected for its demonstrated extraction efficiency of bioactive compounds from Eucalyptus wood industrial wastes [16]. Besides, it is one of the more environmentally friendly and safe extracting agents according to the European Food Safety Authority (EFSA), and Expert Committee of Food Additive/World Health Organization (FAO/WHO) [17].

On the other hand, ethyl lactate is also an environmentally friendly solvent which complies with most of the Green Chemistry principles. Additionally, it is allowed for its use in food products by the United States Food and Drug Administration (FDA) and the European Union, and it is miscible with both hydrophilic and hydrophobic compounds, being a very suitable good option for the reutilization of the obtained extracts in the food industry. Moreover, its effectiveness in the extraction of plant phenolics has already been evaluated by the authors [18].

25 mL of screw water or concentrate of Eucalyptus were mixed with 25 mL of the correspondent solvent (ethanol or ethyl lactate) into a Falcon 50 mL conical centrifuge tube and manually shaken for 1 min. Afterwards, the mixture was centrifuged at 3500 rpm for 10 min employing an Ortoalresa Digicen 21centrifuge (Madrid, Spain). One mL of the organic supernatant was transferred to a 1.8 mL glass vial, and Na_2_SO_4_ was added to remove possible aqueous content. Finally, the dried extract was filtered through 0.22 µm polytetrafluoroethylene (PTFE) filters, and directly analyzed by GC-MS or LC-MS/MS.

### 2.4. Total Polyphenols Content and Antioxidant Activity Procedures

The total polyphenols content (TPC) of the raw wood by-products and their derived organic extracts were determined according to the Folin–Ciocalteu (FC) colorimetric method described by Singleton and Rossi [19]. The TPC was quantified employing a calibration curve prepared with gallic acid standards solutions ranging from 3 to 20 mg L^−1^ (*R*^2^ = 0.9970) and expressed as mg of gallic acid equivalents in the liquid extract (mg GAE L^−1^).

The antioxidant activity (AA) was determined employing a modified method of Brand-Williams et al. [20]. 0.1 mM DPPH was dissolved in methanol. 100 mL of the different wood by-products studied (both raw materials and their derived organic extracts) were added to 3.9 mL of the methanolic DPPH solution. The mixture was vortexed assisted shaken and kept at room temperature in the dark for 30 min. Afterwards, the absorbance was measured at 515 nm employing a Shimazdu UVmini-1240 Spectrophotometer (Kyoto, Japan). The AA was calculated employing a calibration curve prepared with Trolox ranging from 0.1–1 mM (*R*^2^ = 0.9994). The DPPH scavenging activity is expressed as mM Trolox equivalents in the liquid extract (mM TRE L^−1^).

### 2.5. Solid-Phase Microextraction Procedure

Aliquots of 10 mL of the corresponding by-products (screw water, condensates or concentrate of Eucalyptus) were placed in a 22 mL glass vial. The vials were sealed with aluminium caps furnished with PTFE-faced septa and immersed into a water bath maintained at 100 °C under magnetic stirring. After 5 min of sample equilibration, the PDMS/DVB fibre was introduced into the vial and exposed to the headspace over the sample for 30 min. Afterwards, the fibre was retracted into the needle of the holder syringe, thermally desorbed in the GC injector for 5 min and GC-MS analysis was carried out.

### 2.6. GC-MS Analysis

The GC-MS analysis was performed using an Agilent 7890A coupled to an Agilent 5975C inert mass spectra detector (MSD) with triple-axis detector and an Agilent 7693 autosampler from Agilent Technologies (Palo Alto, CA, USA). Two different columns were employed: a ZB-Semivolatiles (30m × 0.25 mm i.d., 0.25 µm film thickness) column obtained from Phenomenex (Torrance, CA, USA), with a chromatographic ramp that implies 60 °C (held 1 min) to 290 °C at 5 °C min^−1^ (held 1 min) (total run time: 48 min) or a polar DBWAX column (50 m × 0.20 mm i.d., 0.20 µm film thickness) obtained from Agilent Technologies. In this case, the GC oven temperature was programmed from 50 °C (1 min) to 240 °C at 8 °C min^−1^ (held 25.25 min) (total run time: 50 min). In both cases, helium (purity 99.999%) was employed as carrier gas at a constant flow of 1.0 mL min^−1^ (non-polar column) or 0.6 mL min^−1^ (polar column). The sample volume was 1 µL when direct injection was performed (organic extracts). The injector temperature was 270 °C, and 240 °C, for the non-polar, and polar columns, respectively. In both cases, the mass spectrometer detector (MSD) was operated in the electron impact (EI) ionization positive mode (+70 eV), and the temperatures of the transfer line and the ion source were set at 230 °C and 150 °C, respectively.

Full Scan (FS) acquisition mode was employed, monitoring mass/charge (*m/z)* fragments between 25–700. The identification of the compounds was performed by comparison (match > 80%) between the obtained experimental MS spectral and those provided by the spectral library database (NIST).

### 2.7. LC-MS/MS Analysis

The identification and quantification of the polyphenols was performed by LC-MS/MS employing a Thermo Scientific (San José, CA, USA) instrument based on a TSQ Quantum Ultra TM triple quadrupole mass spectrometer equipped with a HESI-II (heated electrospray ionization), and an Accela Open autosampler with a 20 µL loop. The chromatographic separation was achieved on a Kinetex C18 column (100 × 2.1 mm, 2.6µm, 100 Å), obtained from Phenomenex. The temperature of the column was set at 50 °C. The mobile phase consisted on water (A) and methanol (B), both with 0.1% formic acid. The eluted gradient started with 5% of B, it was increased to 90% of B in 11 min and kept constant for 3 min. Finally, initial conditions were reached in 9 min. The injection volume was 10 µL and the mobile phase flow-rate was 0.2 mL min^−1^. The total run for each injection was 25 min. The mass spectrometer and the HESI source were working simultaneously in the positive and negative mode, monitoring two or three MS/MS transitions for each compound. MS/MS transitions for the identified polyphenols are summarized in Table S1. For quantification purposes, calibration curves were prepared in water for each compound covering a concentration range between 0.1–10 mg L^−1^. In all cases, good linearity with coefficients of determination (R^2^) higher than 0.9952 were obtained (see Table S1).

## 3. Results

For all the studied Eucalyptus wood industry by-products (screw water, condensates, and concentrate) and for the Eucalyptus screw water- and concentrate-derived organic extracts, basic physico-chemical parameters, such as density and pH were determined. Besides, the total polyphenols content (TPC) and antioxidant activity (AA) were measured. The obtained values are summarized in Table 1.

### 3.1. Eucalyptus Screw Water

**Figure 2:**
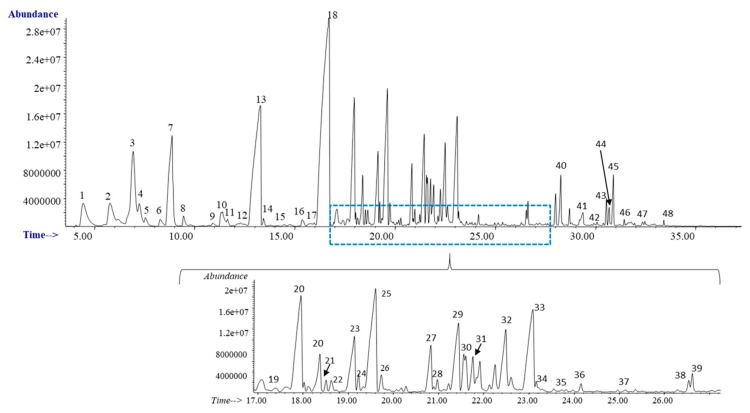
Chromatographic profile obtained for the Eucalyptus screw water. SPME-GC-MS analysis.

The Eucalyptus screw water was directly characterized by SPME-GC-MS (non-polar column). Besides, derived organic extracts in ethanol and ethyl lactate were obtained and directly analyzed by GC-MS (polar column). Figure 2 shows the chromatographic profile (non-polar column) for the Eucalyptus screw water, and the identified compounds are summarized in Table 2. As can be seen, up to 48 different organic compounds were detected in the Eucalyptus screw water, being the most abundant compounds trans-geraniol (Peak 13), and two of its derivates, geranyl acetate (Peak 18) and geranyl butyrate (Peak 25). Besides, the presence of other compounds with interesting antimicrobial, antifungal and anti-inflammatory properties highlights, such as monoterpenes (α-Pinene (Peak 1), myrcene (Peak 2), γ-Terpinene (Peak 5), allo-ocimene (Peak 6) and one of its derivatives (Peak 8)), oxygenatedmonoterpenes (eucalyptol (Peak 3)), terpineol derivatives (Peaks 9,10), sesquiterpenes (alloaromadendrene (Peak 20), ledene (Peak 23), α-Selinene (Peak 24), β-Cadinene (Peak 26)), and oxygenated sesquiterpenes ((−)-Globulol (Peak 29), β-Eudesmol (Peak 33)), among others [21,22]. Several of these compounds have been previously identified in Eucalyptus globulus fruits and leaves [23,24].

**Figure 3:**
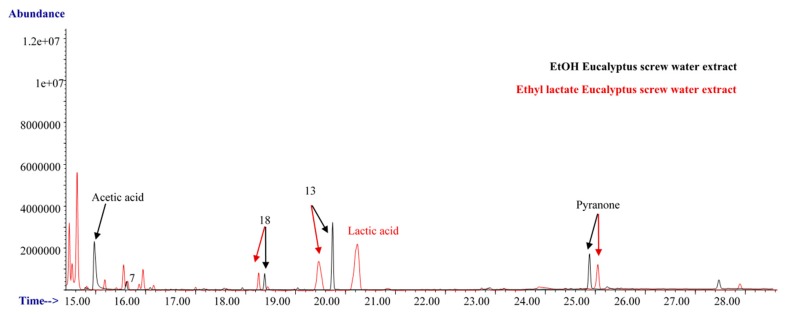
Chromatographic profile for the ethanolic (black) and ethyl lactate (red) extracts derived from the screw water. GC-MS analysis. The differences in the retention times are due to the different organic solvents employed.

The chromatographic profiles (polar column) for the organic extracts, ethanolic (black) and ethyl lactate (red), derived from the Eucalyptus screw water are shown in Figure 3, and the identified compounds are also summarized in Table 2. As can be seen, 3 of the compounds (linalol (Peak 7), trans-geraniol (Peak 13), and geranyl acetate (Peak 18)), detected in the screw water, were also identified in its derived organic extracts. Besides, as can be seen in Figure 3, two acids, acetic and lactic acid were detected in the ethanolic and in the ethyl lactate derived extracts, respectively, whereas pyranone was found in both extracts.

### 3.2. Eucalyptus Condensates

**Figure 4:**
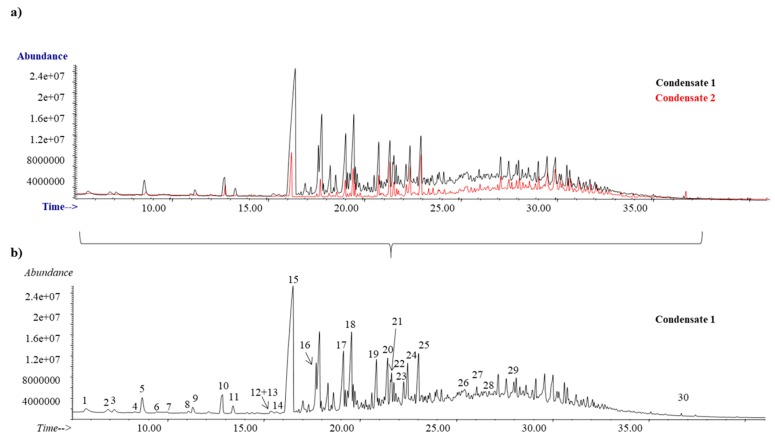
Chromatographic profile for (**a**) Condensate 1 (black) and Condensate 2 (red) and (**b**) detailed characterization for Condensate 1. SPME-GC-MS analysis.

As discussed in Section 2.2, two different types of condensates samples (Condensate 1 and Condensate 2) were collected during the industrial process (see Figure 1), and both were characterized by SPME-GC-MS (non-polar column, see detailed experimental procedure in Section 2.5). The chromatographic abundance for Condensate 1 (black) and Condensate 2 (red) is shown in Figure 4a, being their chromatographic profiles similar in both cases. However, the abundance for most of the compounds was clearly higher in the Condensate 1 in comparison with Condensate 2, and for that reason Figure 4b shows the detailed chromatogram for this last one, where up to 30 different compounds were clearly identified, and they are summarized in Table 3. The two most abundant compounds were: geranyl acetate (Peak 15) and geranyl butyrate (Peak 18). They are recognized as safe substances, allowed for their use as synthetic flavouring and, they are also usually employed in the cosmetic industry as perfuming agents. It is important to highlight that although both condensate samples showed a very low TPC (see values in Table 1), the presence of fragrances and other flavouring agents allows the reutilization of this wood industry by-product as green additive in food, beverages or cosmetics.

### 3.3. Concentrate of Eucalyptus

The density, pH, TPC and AA values for the concentrate of Eucalyptus are summarized in Table 1. As can be seen, the TPC and AA values were clearly higher in comparison with those obtained for the other studied by-products (screw water and condensates).

SPME-GC-MS analysis (non-polar column) was performed for the raw material (see detailed procedure in Section 2.5), and 12 different organic compounds have been successfully identified including sesquiterpenes (ledene, alloaromadendrene), oxygenated sesquiterpenes (α-eudesmol), fragrances and food additives (geraniol butyrate), compounds with a demonstrated high antibacterial and antifungal activity (mellein, antiarol), several saturated fatty acids (lauric acid, myristic acid, palmitolinoleic acid, palmitic acid), and other compounds usually identified as constituents of Eucalyptus globulus essential oils (*n*-heptadecane, *n*-nonadecane) [3,6,10].

However, unlike the other by-products, the direct use of the concentrate was much more complex, because it has residual fibres of such a small size that was very difficult to remove by filtration. For this reason, a liquid–liquid extraction strategy was proposed to obtain an extract enriched in the bioactive compounds of the original concentrate and simultaneously, completely free of fibres. As a matter of fact, the extraction procedure produced a homogeneous precipitate of fibres with a very operable consistency, that is very easy to handle and isolate, and in addition, it can be reused in a complementary way in the manufacturing process of the greenboards improving their properties. This approach has been demonstrated on a laboratory scale so far, but the scaling-up is being assessed due to its promising performance. Therefore, organic extracts in ethanol and ethyl lactate, derived from the concentrate of Eucalyptus, were also obtained. The detailed procedure was previously described in Section 2.3. The density, pH, TPC and AA results are shown in Table 1. The high values, keeping more than 50% on average of the raw material scores indicate that, a priori, several compounds with antioxidant properties could be present in the organic extracts along with the above mentioned organic compounds. For that reason, both ethanolic and ethyl lactate-based extracts were characterized not only employing GC-MS, but also LC-MS/MS in order to identify the presence of polyphenols, very recognized compounds with a high antioxidant activity among other interesting bioactivities, which could give an added value to the extracts obtained from the Eucalyptus concentrate.

**Figure 5:**
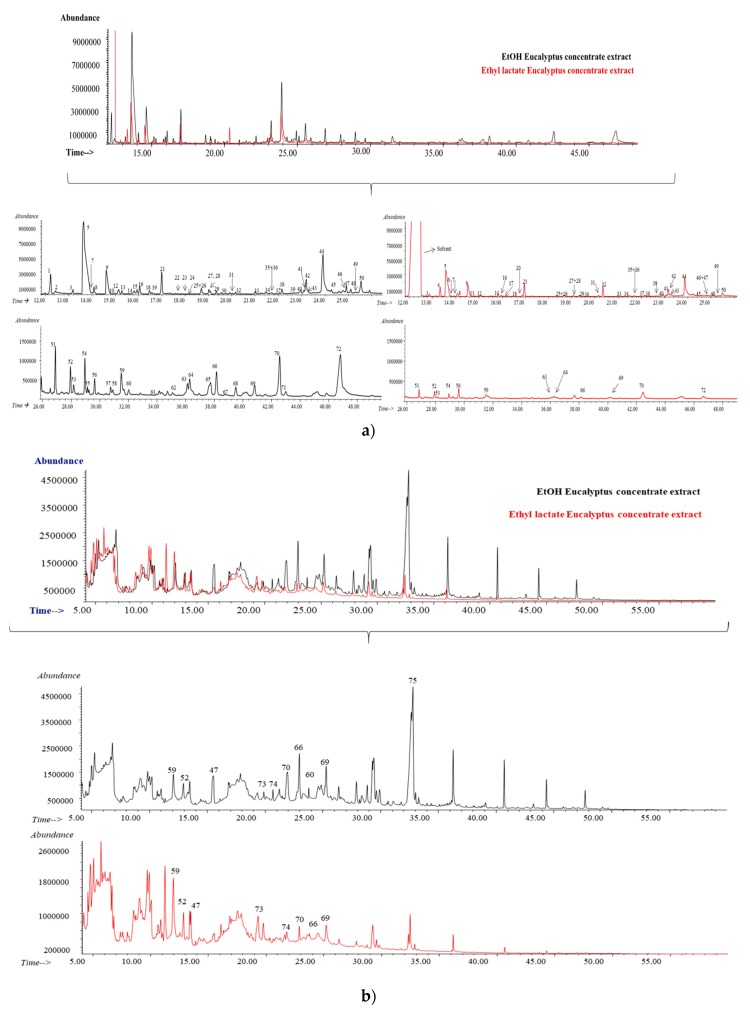
Chromatographic profile obtained for the ethanolic (black) and ethyl lactate (red) extracts derived from the concentrate of Eucalyptus in the (**a**) polar column and (**b**) non-polar column.

For the characterization of the ethanolic and ethyl lactate extracts, GC-MS analysis employing both polar and non-polar chromatographic columns was used to obtain an exhaustive screening. Figure 5a,b show the obtained chromatographic profile employing the polar and non-polar column for the ethanolic (black) and for the ethyl lactate (red) extracts, respectively. As can be seen, they were slightly different, showing the ethanolic extract not only a high number of compounds, but also a higher abundance for the same compounds which were identified in the ethyl lactate-based extract, employing the polar (Figure 5a) and the non-polar chromatographic column (Figure 5b). Table 4 summarizes all the identified compounds. As can be seen in Figure 5a, up to 72 different compounds were detected when the GC-MS analysis was performed employing the polar column. Sixty-seven out of the 72 were found in the ethanolic extract, whereas 52 were detected in the ethyl lactate-based extract. Among the identified compounds, highlights the abundance of acetic acid (Peak 5), and methyltartronic acid (Peak 44) in both organic derived extracts. This last one results from alkaloids degradation, and it has several applications in the pharmaceutical industry [25]. It is also important to note the presence of 5-hydroxymethylfurfural (Peak 52) in both organic extracts. This compound is generated during the dehydration of carbohydrates. 5-hydroxymethylfurfural is deemed as a key intermediate between biomass and biochemicals, since it is considered to be a natural and toxin-free formaldehyde replacement. It contains an aldehyde group and an alcohol functional group, which allows several structural possibilities once broken into furan-monomers, leading more than 175 product derivatives. These furans have been called the sleeping giants of renewable chemicals due to their high potential [26]. Indeed, several derivatives have been detected in the characterized extracts (2-furfuryl-acetate (peak 10), 2-furfurylmethyl ketone (Peak 11), furfuryl alcohol (Peak 21)), and especially furfural (peak 8), used as a solvent or as an extraction agent, which can be converted in new families of bio-based, sustainable chemicals or fuel [27]. In fact, it has been included among the top 30 added-value chemicals from biomass [28], and it has been also detected in Eucalyptus leaves and wood [24,27].

Other detected compound with interesting properties was propanoic acid (Peak 12), which is frequently used as preservative for both animal feed, and food for human consumption and it is also useful as an intermediate in the production of polymers like cellulose [29]. Phenolic aldehydes such as vanillin (Peak 55), its derivative vanillyl methyl ketone (Peak 57), and the dimethyl ether syringol (peak 47) have been identified. The presence of these compounds is related with the thermal decomposition of lignine [13,14].

Regarding the analysis employing the non-polar column (Figure 5b), up to 10 compounds were successfully identified (see Table 4) in the ethanolic extract, whereas seven were found in the extract of ethyl lactate. Several of the detected compounds were also found employing the polar column, such as syringol (Peak 47), 5-hydroxymethylfurfural (Peak 52), catechol (Peak 59), methoxyeugenol (Peak 60), syringaldehyde (Peak 66), desaspidinol (Peak 69) or antiarol (Peak 70). On the other hand, the use of the non-polar column allowed the identification of 6-hydroxycoumarin (Peak 73), and acetoguaiacon (Peak 74) in both organic extracts whereas linolenic acid (Peak 75), the most abundant peak, was only detected in the ethanolic extract.

In view of the high TPC and AA values for the ethanolic and ethyl lactate extracts derived from the concentrate of Eucalyptus, their polyphenolic profile was determined by LC-MS/MS. Due to the absence of spectral libraries such as those of GC-MS, the identification was performed based on the MS/MS transitions, and retention time of the standard compounds, and therefore limited to the standards available in the laboratory. Table 5 shows the quantification of the compounds detected in the extracts (their MS/MS transitions and retention times are shown in Table S1). As can be seen, up to five different polyphenols were found, highlighting the presence of gallic acid in both extracts, with concentrations up to 226 mg L^−1^ in the ethanolic-based extract. Other polyphenols detected in both extracts were protocatechuic acid and chlorogenic acid with concentrations ranging between 19–26 mg L^−1^ and 0.4–0.6 mg L^−1^, respectively. On the other hand, 3,4-dihydroxybenzaldehyde and 4-hydroxybenzaldehyde were only detected in the ethanolic extract with concentration values between 0.6–1.3 mg L^−1^. The presence of two known compounds possessing high radical scavenging activity, such as gallic and chlorogenic acids have been previously identified in several parts of the Eucalyptus tree, such as leaves [30] or bark [31].

#### Stability Studies

The concentrate of Eucalyptus is the main by-product generated during the industrial process in terms of volume of production, and for its physico-chemical properties, especially for its high density (see values in Table 1), its reuse could be a priori difficult. However, in view of the results, it showed the highest TPC and AA values (see Table 1), and a higher number of organic volatile compounds were also detected. Therefore, for a practical reutilization of the concentrate of Eucalyptus and/or its derived extracts in the food or cosmetic industry, it is important to ensure the stability of the raw material over time. In this way, the concentrate of Eucalyptus was stored protected from light at three different temperatures (−20 °C, 4 °C and 25 °C) for 1 year, and the pH, TPC and AA were periodically (weekly for the two first months, and then monthly for one year) measured. As can be seen in Table 1, the initial pH of the raw material was 3.69. After 1 year, the average pH values were 3.77, 3.74, and 3.68 for −20 °C, 4 °C and 25 °C, respectively. Regarding the TPC and AA, the mean values at the three evaluated temperatures for the studied period were 43301 ± 439 mg GAE L^-1^, and 194 ± 2 mM TRE L^−1^, respectively. Statistical analysis based on analysis of variance (ANOVA) were performed and no significant statistical differences (*p*-value > 0.05 denotes no statistical significance) were observed for the three studied parameters (pH: *F* = 1.2, *p*-value = 0.3168; TPC: *F* = 0.56, *p*-value = 0.5770; AA: *F* = 0.48, *p*-value = 0.6251). Therefore, it has been demonstrated that the concentrate of Eucalyptus is stable in terms of pH, TPC and AA at three different temperatures for one year, showing its suitability for its reutilization in several industries.

## 4. Conclusions

Significant differences have been observed in the obtained chromatographic profiles for the three studied by-products. When GC-MS analysis was carried out, up to 48 and 30 different volatile organic compounds from different chemical nature have been identified in the *Eucalyptus* screw water, and condensates, respectively, whereas up to 75 different compounds were found in the concentrate, and in their derived ethanolic and ethyl lactate extracts. The research highlights the presence of terpenes, sesquiterpenes, omega-3 fatty acids, and precursors of fragrance synthesis. The LC-MS/MS analysis allowed the identification of five different polyphenols in the organic extracts derived from the concentrate of Eucalyptus. This analysis highlights the presence of gallic acid in the ethanolic and ethyl lactate-based extracts at concentration levels up to 226 mg L^−1^. Most of the identified compounds present antioxidant, antimicrobial, antifungal and interesting organoleptic properties, demonstrating that these industrial wastes could be an interesting option for their reuse in the food, pharmaceutical and/or cosmetic industry, reducing the environmental impact of the wood industry activity and obtaining, in parallel, an economical profit.

## Figures and Tables

**Table 1:** Density, pH, TPC and AA values for the studied by-products and their derived extracts.

By-Product		Density (g mL^−1^)	pH	TPC (mg GAE L^−1^)	AA (mM TRE L^−1^)
Eucalyptus screw water	Raw material	0.9870	3.65	6652 ± 21	52 ± 1
	EtOH extract	0.9928	4.27	3630 ± 66	33 ± 1
	Ethyl lactate extract	1.002	4.03	4005 ± 49	34 ± 1
Condensates	1	1.0020	2.97	6.1 ± 0.06	----
	2	1.0042	2.89	7.7 ± 0.2	----
Concentrate of Eucalyptus	Raw material	1.1059	3.69	43,702 ± 1413	197 ± 1
	EtOH extract	0.9336	4.74	17,001 ± 377	101 ± 3
	Ethyl lactate extract	1.0320	4.32	30,287 ± 417	165 ± 6

**Table 2:** Identified compounds in the Eucalyptus screw water (SPME-GC-MS analysis), and in its derived organic extracts (GC-MS).

Code	Compound	Ret. Time (min)	CAS	Screw Water (SPME-GC-MS)	Screw Water Extracts	
					EtOH	Ethyl Lactate
1	α-Pinene	4.46	7785-70-8	X		
2	Myrcene	5.79	123-35-3	X		
3	Eucalyptol	6.94	470-82-6	X		
4	β-Ocimene	7.26	13877-91-3	X		
5	γ-Terpinene	7.56	99-85-4	X		
6	Allo-ocimene	8.35	673-84-7	X		
7	Linalol	8.82	78-70-6	X	X	X
8	Neo-allo-ocimene	9.82	7216-56-0	X		
9	4-Terpineol	10.95	562-74-3	X		
10	α-Terpineol	11.4	98-55-5	X		
11	Isoamyl caproate	11.69	2198-61-0	X		
12	Cis-Geraniol	12.34	106-25-2	X		
13	Trans-Geraniol	13.26	106-24-1	X	X	X
14	α-Citral	13.46	141-27-5	X		
15	Bornyl acetate	13.82	76-49-3	X		
16	α-Terpineol acetate	15.4	80-26-2	X		
17	Nerol acetate	15.79	141-12-8	X		
18	Geranyl acetate	16.52	105-87-3	X	X	X
19	Caryophyllene	17.38	87-44-5	X		
20	Alloaromadendrene	17.91/18.41	25246-27-9	X		
21	Butanoic acid, 2-methyl-, 3,7-dimethyl-2,6-octadienyl ester, (E)	18.51	68705-63-5	X		
22	2-Isopropenyl-4a,8-dimethyl-1,2,3,4,4a,5,6,7 -octahydronaphthalene	18.63	192435 (NIST Number)	X		
23	Ledene	19.12	21747-46-6	X		
24	α-Selinene	19.22	473-13-2	X		
25	Geranyl butyrate	19.54	106-29-6	X		
26	β-Cadinene	19.73	523-47-7	X		
27	Ledol	20.81	577-27-5	X		
28	1,2-Dimethyl-5-nitroadamantane	20.9	6588-68-7	X		
29	(−)-Globulol	21.38	489-41-8	X		
30	Viridiflorol	21.57	552-02-3	X		
31	Butanoic acid, 2-methyl-, 3,7-dimethyl-2,6-octadienyl ester, (Z)-	21.76	68705-63-5	X		
32	1H-Indene, 1-ethylideneoctahydro-7a-methyl-, (1Z,3aα,7aβ)-	22.24	56324-69-7	X		
33	β-Eudesmol	22.99	473-15-4	X		
34	Guai-1(10)-en-11-ol	23.16	22451-73-6	X		
35	Geranyl hexanoate	23.97	10032-02-7	X		
36	Farnesol	24.15	4602-84-0	X		
37	Myristic acid	25.12	544-63-8	X		
38	2-Propanone, 1-methyl-1-(2,4,6-trimethoxyphenyl)	26.54	26537-69-9	X		
39	Farnesol acetate	26.62	352672 (ID)	X		
40	2-Propanone, 1-methyl-1-(2,4,6-trimethoxyphenyl)	28.21	26537-69-9	X		
41	Hexadecanoic acid	29.3	57-10-3	X		
42	Ethyl hexadecanoate	29.8	628-97-7	X		
43	3,5-di-tert-Butyl-4-hydroxycinnamic acid	30.5	22014-01-3	X		
44	Falcarinol	30.66	21852-80-2	X		
45	6,11-dimethyl-2.6,10-dodecatrien-1-ol	30.89	196533 (NIST number)	X		
46	Quinazoline, 3-4-dihydro-2-allylthio-4-spirocyclohexane	31.42	271789 (NIST number)	X		
47	Linoleic acid	32.31/32.45	60-33-3	X		
48	Geranyl linalool	33.39	1113-21-9	X		

**Table 3:** Identified compounds in the Condensate 1 sample. SPME-GC-MS analysis.

Code	Compound	Ret. Time (min)	CAS
1	β-Myrcene	5.78	123-35-3
2	Eucalyptol	6.91	470-82-6
3	β-cis-Ocimene	7.26	3338-55-4
4	α-Terpinene	8.33	99-86-5
5	β-Linalool	8.62	78-70-6
6	Trans-allocimene	9.46	7216-56-0
7	3,5,5-trimethyl-hexanoic acid	10	3302-10-1
8	Creosol	11.06	93-51-6
9	α-Terpineol	11.28	98-55-5
10	Geraniol	12.81	106-24-1
11	Decan-1-ol	13.39	112-30-1
12	α-Terpinyl acetate	15.37	80-26-2
13	Citronellol acetate	15.44	150-84-5
14	Eugenol	15.45	97-53-0
15	Geranyl acetate	16.18	105-87-3
16	2,6-Di-tert-butylphenol	17.52	128-39-2
17	(+)-Ledene	18.98	21747-46-6
18	Geranyl butyrate	19.38	106-29-6
19	Epiglobulol	20.73	150051 (NIST number)
20	(−)-Globulol	21.3	489-41-8
21	Guaiol	21.52	489-86-1
22	Geranyl-2-methylbutyrate	21.66	68705-63-5
23	1H-Indene, 1-ethylideneoctahydro-7a-methyl-, (1Z,3aα,7aβ)-	22.26	56324-69-7
24	γ-Eudesmol	22.35	1209-71-8
25	β-Eudesmol	22.88	473-15-4
26	Myristic acid	25.26	544-63-8
27	Farnesyl acetate	26.64	4128-17-0
28	4-Methyldibenzothiophene	27.16	7372-88-5
29	3-Ethyldibenzothiophene	28.94	89817-03-8
30	Octadecanoic acid, butyl ester	36.68	123-95-5

**Table 4:** Identified compounds in the ethanolic and ethyl lactate extracts obtained from the concentrate of Eucalyptus. GC-MS analysis.

Code	Compound	Ret. Time (min)	CAS	Polar Column		Non-Polar Column		
				EtOH	Ethyl Lactate	Ret. Time (min)	EtOH	Ethyl Lactate
1	Hydroxyacetone	12.22	116-09-6	X				
2	1-Methoxyacetone	12.44	5878-19-3	X				
3	1-Hydroxy-2-butanone	12.89	5077-67-8	X	X			
4	Butanoic acid, 2-hydroxy-, ethyl ester	13.16	52089-54-0		X			
5	Acetic acid	13.67	64-19-7	X	X			
6	Acetoxyacetone	13.93	592-20-1		X			
7	Methyl pyruvate	13.99	600-22-6	X	X			
8	Furfural	14.1	98-01-1	X	X			
9	Formic acid	14.6	64-18-6	X	X			
10	2-Furfuryl-acetate	14.99	623-17-6	X				
11	2-Furyl methyl ketone	14.8	1192-62-7		X			
12	Propanoic acid	15.16	79-09-4	X				
13	2,3-Butanediol	15.3	24347-58-8	X				
14	5-Methyl-2-furfural	15.8	620-02-0	X	X			
15	Vinyl 2-butenoate	15.9	14861-06-4	X				
16	Propylene glycol	16.06	57-55-6	X	X			
17	4-Cyclopentene-1,3-dione	16.08	930-60-9		X			
18	Butanoic acid	16.4	107-92-6	X	X			
19	Ethylene glycol	16.59	107-21-1	X				
20	4-hydroxy-butanoic acid	16.87	591-81-1		X			
21	Furfuryl alcohol	17	98-00-0	X	X			
22	2-Cyclopenten-1-one, 3-ethyl-2-hydroxy-	17.61	21835-01-8	X				
23	5-Methyl-2-furfuryl alcohol	17.88	3857-25-8	X				
24	Pentanoic acid	18.05	109-52-4	X				
25	2(5H)-Furanone	18.66	497-23-4	X	X			
26	2-Cyclopenten-1-one, 2-hydroxy-	18.7	10493-98-8	X	X			
27	1-(2-Butoxyethoxy)ethanol	19.03	54446-78-5	X	X			
28	1,2-Diisopropylhydrazine	19.1	3711-34-0	X	X			
29	Furfuryl alcohol	19.31	98-00-0	X	X			
30	Cyclotene	19.55	80-71-7	X	X			
31	2,5-Dimethyl-4-hydroxy-3(2H)-furanone	20.02	3658-77-3	X	X			
32	N-Butyl-tert-butylamine	20.17	16486-74-1	X	X			
33	Glycerin	21	56-81-5	X	X			
34	Maltol	21.48	118-71-8	X	X			
35	Glutaconic anhydride	21.68	5926-95-4	X	X			
36	Phenol acetate	21.74	122-79-2	X	X			
37	Furyl hydroxymethyl ketone	22.04	17678-19-2	X	X			
38	Furaneol	22.16	3658-77-3	X	X			
39	2(3H)-Furanone, 5-acetyldihydro-	22.66	29393-32-6	X	X			
40	Dihydroxyacetone	22.97	96-26-4	X	X			
41	Methyl acetoxyacetate	23.1	5837-80-9	X	X			
42	Cyclopropyl carbinol	23.2	2516-33-8	X	X			
43	4,5-Dimethyl-1,3-dioxol-2-one	23.24	37830-90-3	X	X			
44	Methyltartronic acid	23.92	595-98-2	X	X			
45	2-Acetylresorcinol	24.6	699-83-2	X	X			
46	1,1-Ethanediol, diacetate	24.79	542-10-9	X	X			
47	Syringol	24.93	91-10-1	X	X		X	X
48	Pyranone	25.12	28564-83-2	X	X			
49	2,5-Dihydroxypropiophenone	25.33	938-46-5	X	X			
50	Glycerin	25.55	56-81-5	X	X			
51	3-Pyridinol	26.89	109-00-2	X	X			
52	5-Hydroxymethylfurfural	27.96	67-47-0	X	X		X	X
53	1,2-Dihydroxy-3-methoxybenzene	28.19	934-00-9	X	X			
54	1-(2-Furyl)-1,2-ethanediol	28.96	19377-75-4	X	X			
55	Vanillin	29.12	121-33-5	X				
56	Dihydro-4-hydroxy-2-(3H)-Furanone	29.65	5469-16-9	X	X			
57	Vanillyl methyl ketone	30.78	2503-46-0	X				
58	Myristic acid	30.94	544-63-8	X				
59	Catechol	31.53	120-80-9	X	X		X	X
60	Methoxyeugenol	32.07	6627-88-9	X		23.78	X	
61	3-Hydroxy-4-methoxybenzyl alcohol	33.97	6/6/4383	X				
62	Homovanillyl alcohol	35.10	2380-78-1	X				
63	Ethyl β-d-riboside	36.14	126954 (NIST number)	X	X			
64	*n*-Hexadecanoic acid	36.25	57-10-3	X	X			
65	Palmitoleic acid	36.65	373-49-9	X				
66	Syringaldehyde	38.15	134-96-3	X	X	22.87	X	X
67	1,4-Benzenediol, 2-methoxy-	38.77	824-46-4	X				
68	Homovanillic acid	39.53	306-08-1	X				
69	Desaspidinol	40.82	437-72-9	X	X	25.26	X	X
70	Antiarol	42.58	642-71-7	X	X	21.91	X	X
71	*p*-(3-hydroxybutyl)phenol	43.00	501-96-2	X				
72	Oleic Acid	46.81	112-80-1	X	X			
73	6-hydroxycoumarin		2669-94-5			19.72	X	X
74	Acetoguaiacon		498-02-2			21.22	X	X
75	Linolenic acid		60-33-3			32.61	X	

**Table 5:** Concentration of polyphenols (mg L^−1^) in the organic extracts derived from the concentrate of Eucalyptus. LC-MS/MS analysis.

Polyphenols	Concentrate of Eucalyptus	
	EtOH Extract	Ethyl Lactate Extract
Gallic acid	226 ± 15	72 ± 19
Protocatechuic acid	26 ± 7	19 ± 4
Chlorogenic acid	0.6 ± 0.05	0.4 ± 0.02
3,4-dihydroxybenzaldehyde	1.3 ± 0.1	n.d
4-hydroxybenzaldehyde	0.6 ± 0.03	n.d

n.d: Not detected.

## Supplementary Materials

The following are available online at https://www.mdpi.com/2076-3921/8/8/263/s1, Figure S1: Linear range, coefficients of determination (R^2^), retention time and MS/MS transitions for the identified polyphenols in the concentrate of Eucalyptus organic extracts.
