# Supplementary material for: Antioxidants Profiling of By-Products from Eucalyptus Greenboards Manufacture
Source: Antioxidants (Basel). 2019 Aug 1;8(8):263. doi: 10.3390/antiox8080263 (PMC6719899; doi:10.3390/antiox8080263)
Supplement: Supplementary file 1 [file antioxidants-08-00263-s001.pdf]

antioxidants

Supplementary material

Antioxidants profiling of by-products from Eucalyptus greenboards manufacture

Maria Celeiro [1*], J.Pablo Lamas [1], Rosa Arcas [2], Marta Lores [1]

[1] Laboratory of Research and Development of Analytical Solutions (LIDSA), Department of Analytical Chemistry, Nutrition and Food Science. Faculty of Chemistry. E-15782, Campus Vida, Universidade de Santiago de Compostela, Spain; maria.celeiro.monterol@usc.es; juanpablo.lamas@usc.es; marta.lores@usc.es

[2] Betanzos HB, Betanzos, E-15300, A Coruña, Spain; rarcas@betanzoshb.es

* Correspondence: maria.celeiro.montero@usc.es; Tel.: +34-881-814-461 (M.C)

Table S1. Linear range, coefficients of determination (R^2), retention time and MS/MS transitions for the identified polyphenols in the concentrate of Eucalyptus organic extracts.

Polyphenols	CAS	Linear range (mg L-1)	R^2	Retention time (min)	MS/MS transitions (Collision energy, eV)
Gallic acid	149-91-7	0.1-10	0.9965	2.03	169.02 → 125.04 (17)
					169.02 → 153.1 (15)
Protocatechuic acid	99-50-3	0.1-10	0.9975	3.30	152.98 → 109.04 (17)
					152.98 → 91.04 (28)
					152.98 → 108.03 (26)
3,4-dihydroxybenzaldehyde	139-85-5	0.1-10	0.9982	4.47	137.07 → 136.11 (21)
					137.07 → 91.09 (24)
					137.07 → 92.13 (25)
Chlorogenic acid	327-97-9	0.1-10	0.9956	5.41	353.00 → 191.07 (22)
					353.00 → 85.09 (43)
					353.00 → 93.07 (45)
4-hydroxybenzaldehyde	123-08-0	0.1-10	0.9952	5.64	122.97 → 95.04 (13)
					122.97 → 51.1 (36)
					122.97 → 77.96 (20)

Underlined values: Quantification MS/MS transitions
